# Regional Associations of Cortical Thickness With Gait Variability: The Tasmanian Study of Cognition and Gait

**DOI:** 10.1093/geroni/igaa057.750

**Published:** 2020-12-16

**Authors:** Oshadi Jayakody, Monique Breslin, Richard Beare, Velandai Srikanth, Helena Blumen, Michele Callisaya

**Affiliations:** 1 University Of Tasmania, Hobart, Australia; 2 University Of Tasmania, Hobart, Tasmania, Australia; 3 Monash University, Melbourne, Australia; 4 Albert Einstein College of Medicine, New York, New York, United States; 5 Monash University, Frankston, Australia

## Abstract

Gait variability is a marker of cognitive decline. However, there is limited understanding of the cortical regions associated with gait variability. We examined associations between regional cortical thickness and gait variability in a population-based sample of older people without dementia. Participants (n=350, mean age 71.9±7.1) were randomly selected from the electoral roll. Variability in step time, step length, step width and double support time (DST) were calculated as the standard deviation of each measure, obtained from the GAITRite walkway. MRI scans were processed through FreeSurfer to obtain cortical thickness of 68 regions. Bayesian regression was used to determine regional associations of mean cortical thickness and thickness ratio (regional thickness/overall mean thickness) with gait variability. Smaller overall cortical thickness was only associated with greater step width and step time variability. Smaller mean thickness in widespread regions important for sensory, cognitive and motor functions were associated with greater step width and step time variability. In contrast, smaller thickness in a few frontal and temporal regions were associated with DST variability and the right cuneus was associated with step length variability. Smaller thickness ratio in frontal and temporal regions important for motor planning, execution and sensory function and, greater thickness ratio in the anterior cingulate was associated with greater variability in all measures. Examining individual cortical regions is important in understanding the relationship between gray matter and gait variability. Cortical thickness ratio highlights that smaller regional thickness relative to global thickness may be important for the consistency of gait.

